# Severe Retinal Damage and Choroidal Neovascularization Following Accidental Laser Exposure During Hair Removal: A Case Report

**DOI:** 10.7759/cureus.69075

**Published:** 2024-09-10

**Authors:** Mohammad A Abbas, Najah K Mohammad

**Affiliations:** 1 Department of Surgery, Ophthalmology Unit, Ghazi Al-Harriri for Surgical Specialities Hospital, Medical City Complex, Baghdad, IRQ; 2 Department of Ophthalmology, College of Medicine, University of Baghdad, Baghdad, IRQ

**Keywords:** anti-vegf therapy, choroidal neovascularization, laser hair removal, ocular injury, retinal damage

## Abstract

Ocular injuries from cosmetic laser procedures are perhaps more problematic with regard to laser hair removal, given that some of the losses in vision may be significant and even permanent. Protective eyewear, for example, plays a very critical role in preventing such injuries. The following is a case report of a 24-year-old female technician who suffered serious retinal damage and consequent choroidal neovascularization (CNV) following accidental exposure to a laser hair removal device without wearing protective eyewear. She had first presented with a best-corrected visual acuity (BCVA) of 6/6 in the right eye and 6/60 in the left. An initial optical coherence tomography (OCT) had shown outer retinal damage. Within two weeks, she had a decrease of vision to counting fingers at three meters in the left eye, and it was diagnosed as CNV. Anti-vascular endothelial growth factor (VEGF) intravitreal therapy was promptly initiated. This case underlines the vital importance of very strict safety measures and timely intervention to manage laser-induced ocular injury effectively.

## Introduction

Ocular injuries from lasers used for cosmetic hair removal are becoming an issue for dermatologists and ophthalmologists. This treatment modality was in the preliminary phase until the development of red and infrared laser hair removal in recent years, increasing the possibility of ocular damage if necessary protective measures are not taken. A review pointed out that most eye injuries during dermatologic laser treatments occur due to inadequate or absent ocular protection and lead to serious and often preventable complications [1].

Injuries range from anterior segment damage, such as uveitis and iritis, to damage in the posterior segment, like retinal burns and choroidal neovascularization (CNV). It has been reported that more than 60% of ocular injuries recorded in cosmetic laser treatments were because of not using or removing eye protection, suggesting that stricter safety guidelines are indispensable [2]. Other reports revealed how laser hair removal can cause deep burns, with negative consequences on physical and psychological health [3]. Unintentional macular injury after cosmetic laser adjustment has also been recorded, and this probably showed the susceptibility to causing severe visual impairment [4].

In an accidental alexandrite laser injury, CNV was reported, and prompt treatment with intravitreal anti-vascular endothelial growth factor (VEGF) therapy may help in the prevention of vision loss [5]. This shows that the results of such consequences can be extremely terrible and lasting.

She later developed subfoveal CNV, thereby putting across an important message that, with such injuries, safety measures should be strictly followed so that timely intervention can lead to effective management. This report attempts to increase this base of existing knowledge, with a specific note toward safety measures and peculiarities of management of ocular injuries.

## Case presentation

A 24-year-old woman technician in a beauty center had an accidental laser hair removal device discharge while not wearing protective glasses. The hair removal was performed using the CLARITY II™ laser system (Lutronic Medical Systems Germany GmbH, Hamburg, Germany). She presented to the clinic with immediate complaints of blurred vision, pain in the right eye, and photophobia. She presented to the clinic just after the incident, and her best-corrected visual acuity (BCVA) was measured as 6/6 in the right eye and 6/60 in the left eye. The initial optical coherence tomography (OCT) showed classical signs of outer retinal damage, signifying the injury the laser exposure caused. With the severity of the initial findings, follow-up visits were scheduled for her close monitoring.

**Figure 1:**
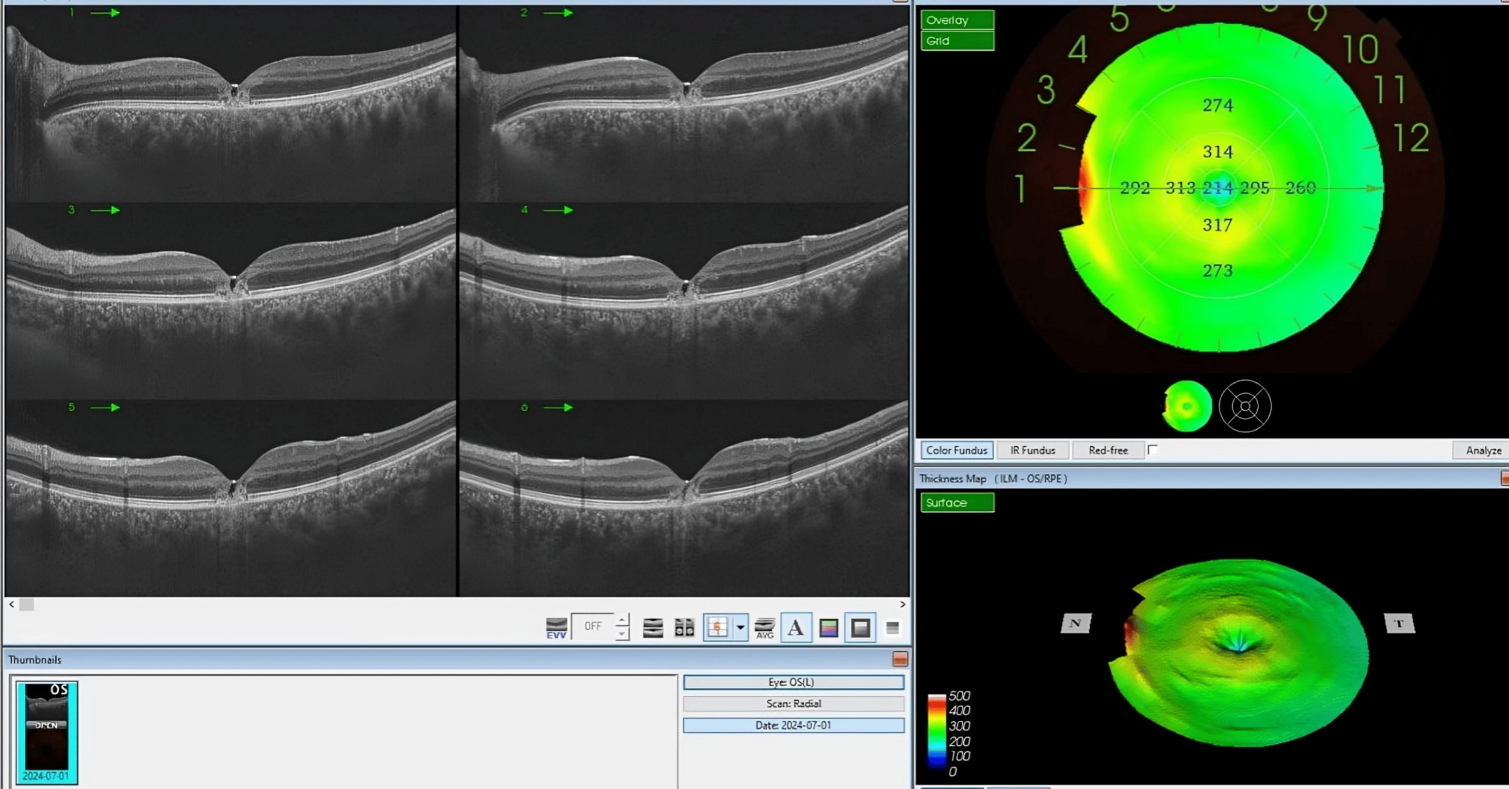
The OCT imaging of the left eye of a 24-year-old female who experienced accidental laser exposure during a hair removal procedure. The cross-sectional scans (left panel) exhibit distinct outer retinal damage, characterized by disruption in the outer retinal layers, particularly at the photoreceptor level, which is indicative of laser-induced retinal injury. There is no evidence of increased thickness or accumulation of subretinal fluid within the images. There is no apparent choroidal neovascularization. A hyperreflective defect is seen underlying the subfoveal RPE, which represents a window defect OCT: optical coherence tomography; RPE: retinal pigment epithelium

**Figure 2:**
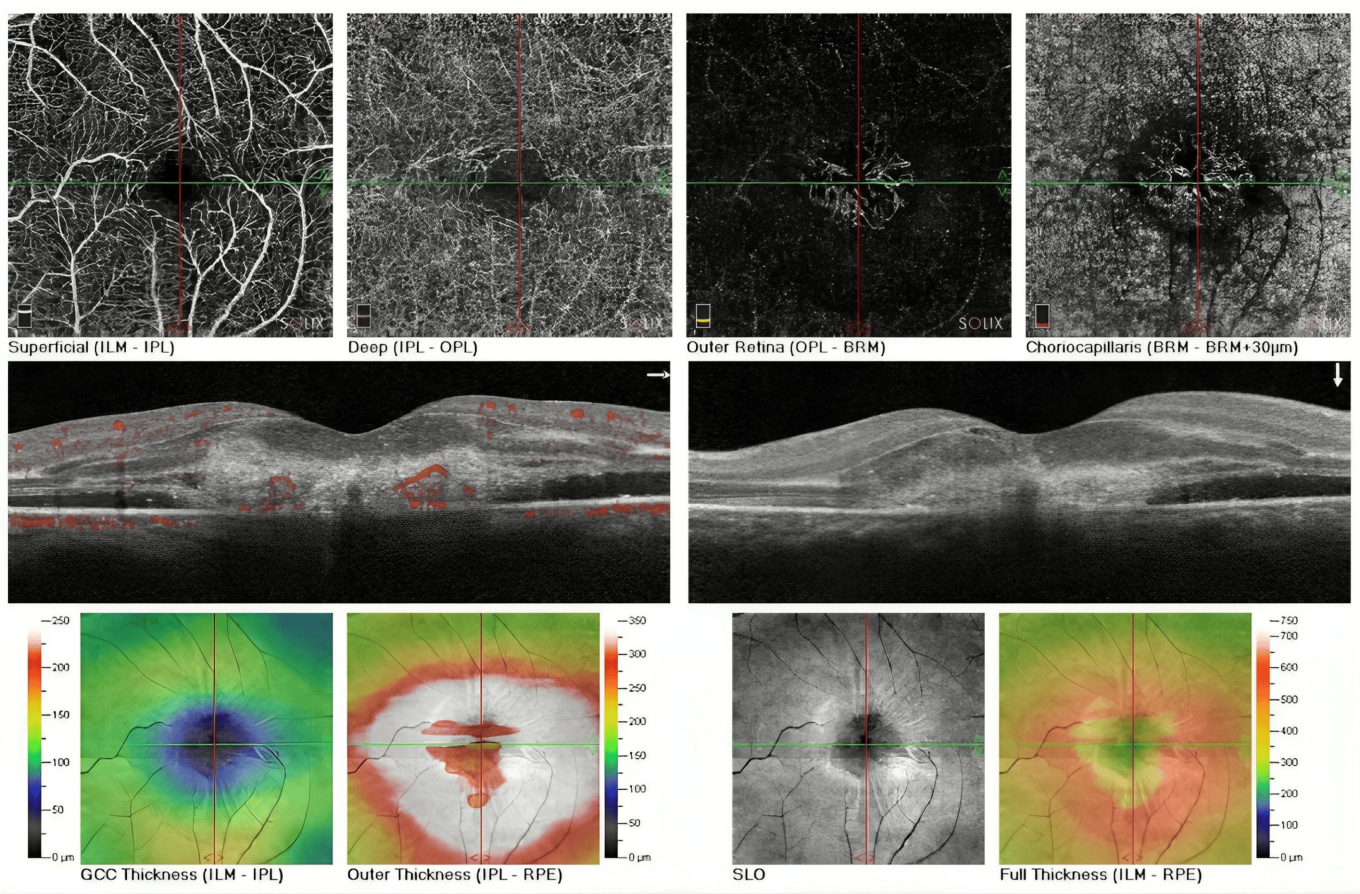
The AngioVue imaging of the left eye of the patient who sustained an accidental laser injury during a hair removal procedure The images of the superficial and deep capillary plexus (top left, center) show a network of vessels without frank abnormality. Images of the outer retina and choriocapillaris show, on the top right, vascular formations with a certain degree of irregularity, with a hyperreflective lesion beneath the RPE that points to possible damage. The cross-sectional OCT-angiography scan (middle) highlights areas of neovascularization with red spots representing abnormal blood flow within the retinal layers. This finding corresponds to the clinical detection of subfoveal CNV, causing the observed visual acuity deterioration. Thickness maps for the GCC and outer retina (bottom left and center) demonstrate areas of thinning and thickening, respectively, with increased thickness centrally correlating with the presence of CNV. The structural en-face images (bottom right) further corroborate these findings, showing the altered retinal architecture and the extent of neovascular involvement. RPE: retinal pigment epithelium; OCT: optical coherence tomography; CNV: choroidal neovascularization; GCC: ganglion cell complex

**Figure 3:**
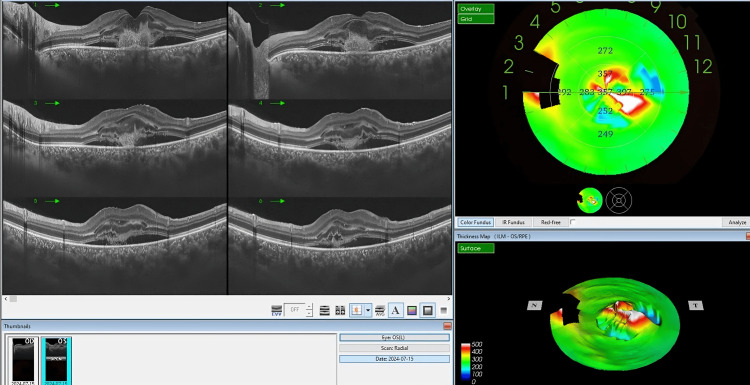
The OCT imaging of the left eye of the patients, two weeks post-accidental laser exposure, shows significant progression in retinal pathology. The B-scan images (left panel) reveal marked changes, including intraretinal and subretinal fluid accumulation, cystoid spaces, and disorganized foveal outer retinal layers due to hyperreflective lesions beneath RPE. The thickness map (top right) demonstrates areas of increased retinal thickness, particularly in the central macula, aligning with the presence of subretinal fluid and cystoid macular edema. The 3D surface map (bottom right) further emphasizes the extent of central macular elevation and distortion, supporting the clinical findings of subfoveal CNV. OCT: optical coherence tomography; RPE: retinal pigment epithelium; CNV: choroidal neovascularization

**Figure 4:**
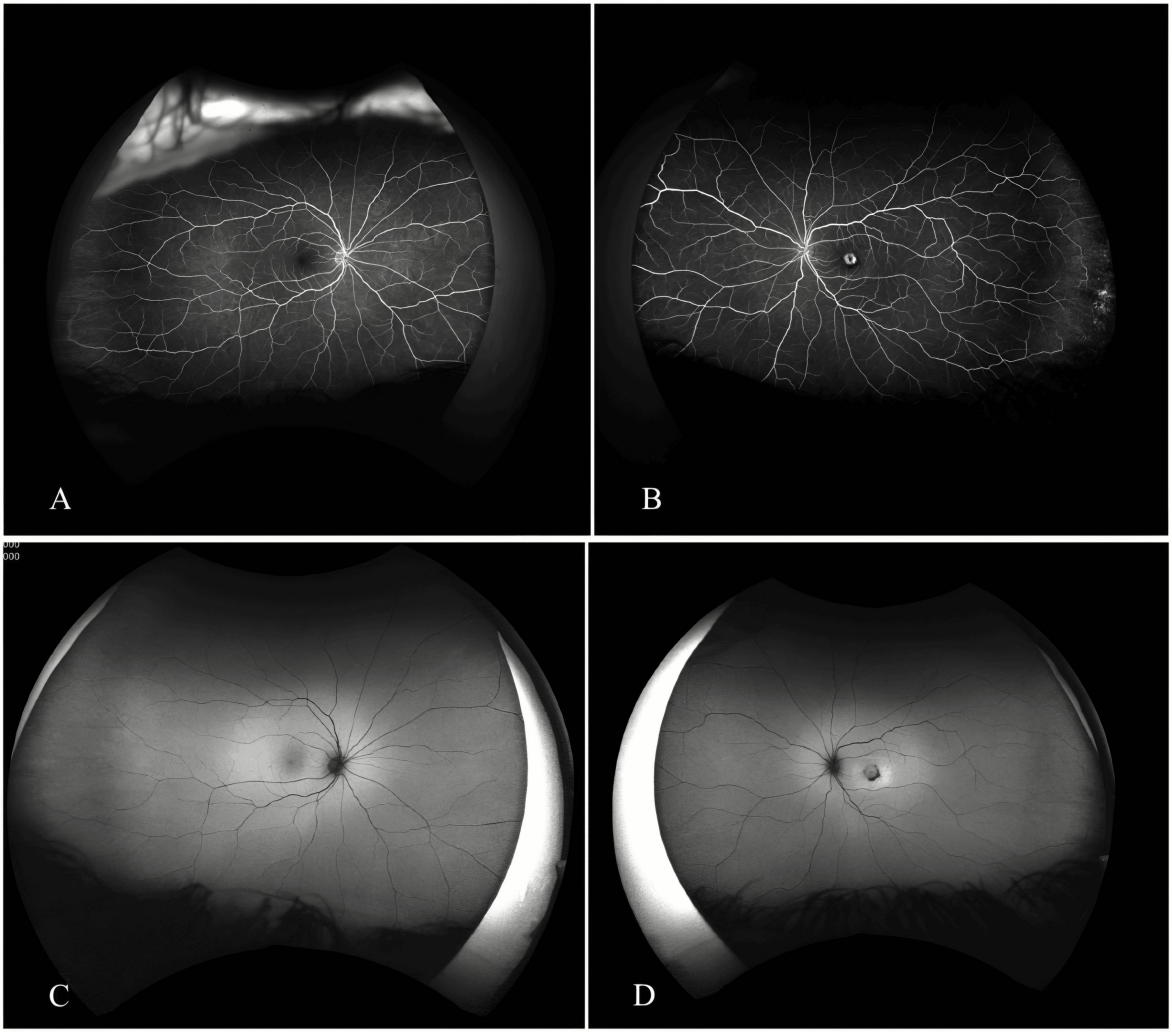
Fluorescein angiography of the left eye A: Early-phase fluorescein angiography of the right eye shows normal retinal vasculature without leakage or significant abnormalities; B: mid-phase fluorescein angiography demonstrates well-defined hyperfluorescence in the macular region, indicative of active leakage from CNV; C: Fundus autofluorescent images of the right eye; D: Fundus autofluorescent images of the left eye. CNV: choroidal neovascularization

Two weeks after the initial presentation, the patient returned to the clinic with complaints of further deterioration in vision in her left eye. On examination, her BCVA had improved substantially to 6/6 in the right eye, and was counting fingers at 3 meters in the left eye, indicating substantial loss of visual function. Subfoveal CNV was observed in the left eye on follow-up (Figures 1-4).

**Figure 5:**
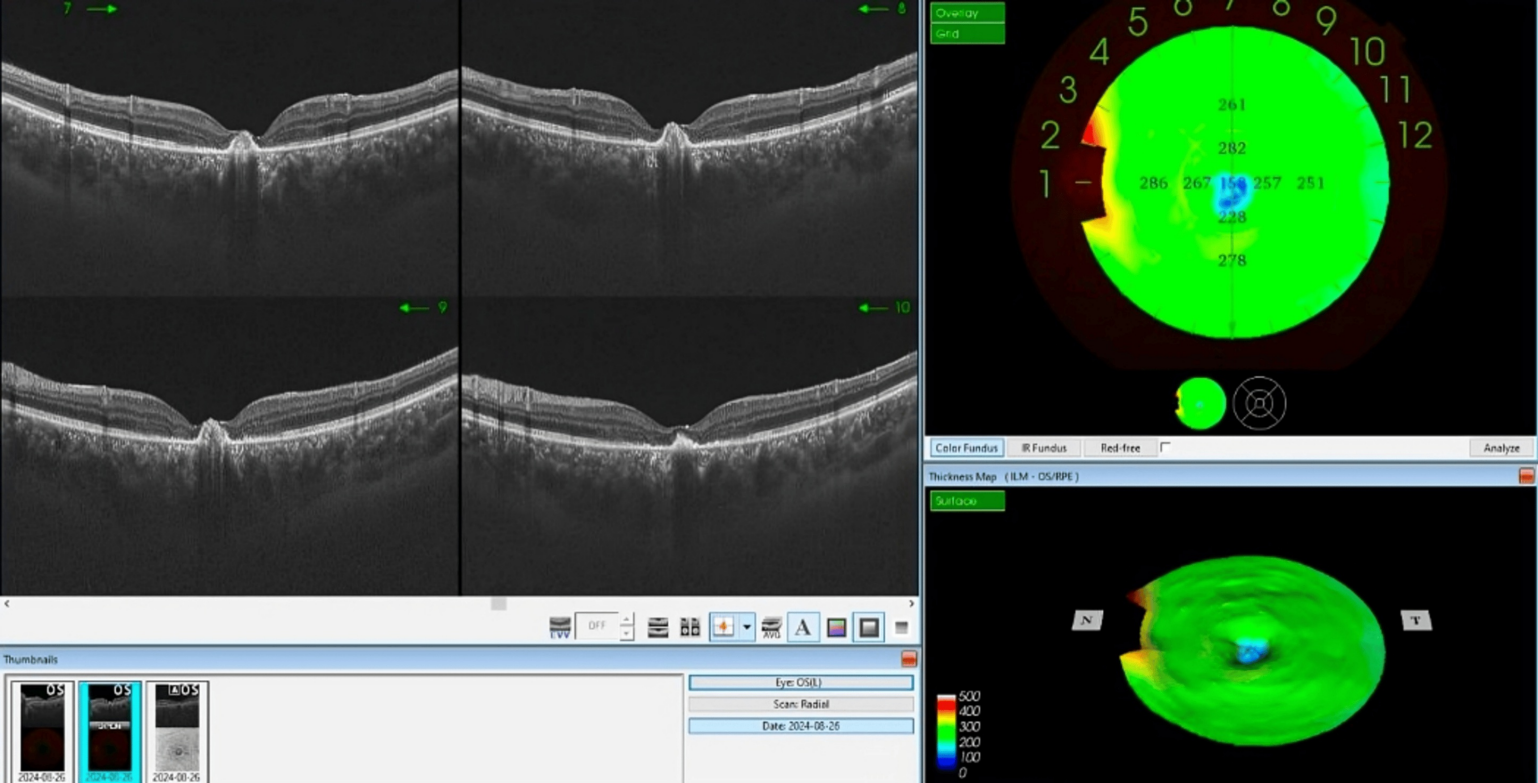
The OCT imaging of the left eye of the patient after two doses of bevacizumab injection The OCT cross-sectional images to the left show disorganization of the foveal outer retinal layers, and thick hyperreflective material underneath the RPE features indicative of active changes in the structure subsequent to the initial trauma and treatment response. While in some ways preserved, there is a disturbance in the contour of the fovea. On the right side of the image, a thickness map is displayed, offering a color-coded representation of the retinal thickness across different regions. The colors on the map range from green to red, with green and yellow indicating areas of normal or slightly thickened retina, while red suggests more significant thickening. Additionally, the lower right portion of the image features a 3D surface map of the retina, which visually represents the variations in retinal thickness. The central area of the retina appears thicker in this map, corroborating the findings seen in the cross-sectional images and thickness map. OCT: optical coherence tomography; RPE: retinal pigment epithelium

This is one of the complications described in the aftermath of severe laser damage to the retina. The patient was given the first dose of anti-VEGF intravitreal therapy promptly to treat the CNV and prevent a further decrease in vision. She was given one injection each month and for two months. The OCT imaging of the left eye of the patient after two doses of bevacizumab injection is showcased in Figure 5.

## Discussion

The rapidly growing cosmetic laser industry, particularly in the field of laser epilation, has unexpectedly brought to light several issues related to ocular safety. Ocular injuries due to dermatologic laser treatments are not infrequent and can vary from mild to severe, with a potential for permanent loss of vision. A discussion of these findings will bring out the need for strict safety procedures and timely treatment.

Ocular laser injuries are often due to the direct impact of the laser beams from failing to use adequate or proper eye protection. Most ocular injuries during dermatologic laser therapy can be preventable with appropriate eye protection. As Flegel et al. [1] found, a considerable proportion of cases had been reported by using unsuitable or no protective eyewear at all, and subsequently damage of different grades to the eyeballs.

In the current case report, a 24-year-old technician had quite a remarkable retinal injury with significant exposure to an inadvertent discharge from a laser hair removal device without wearing protective glasses. The patient's initial BCVA was 6/6 for the right eye and 6/60 in the left eye with severe visual loss. Initial OCT scans showed classical outer retinal damage, common in this type of lesion.

Different types of ocular injuries can occur depending on the laser's wavelength and the part of the eye affected. Huang et al. [2] documented that ocular injuries from cosmetic laser treatments often include anterior segment damage, such as uveitis and iritis, and posterior segment injuries, like retinal burns and CNV. In our case, the technician developed subfoveal CNV two weeks after the initial injury, leading to further deterioration of vision to counting fingers at 3 meters. No other injuries, such as burns or trauma to the skin or face, were reported in this case.

Risk factors are the lack of eyewear protection, insufficient personnel training, and high-fluence use of lasers. Similarly, it was also pointed out by Mallat et al. [6] that injuries to the optical sense are easily preventable and are largely due to professional errors and characteristics of the patients. They suggested that there should be optimal knowledge about laser physics and proper training of the laser operator to minimize the chances of untoward side effects [7,8].

Lack of protective goggles was one of the most important contributing factors responsible for the injury in our case. This seriously points out the importance of strict protocol compliance, including the need for mandatory eye protection for the patient and the operator during all laser procedures. Finally, routine training and certification of employees who handle laser equipment would greatly help reduce the incidence of these injuries.

Early and proper treatment is also another step in handling injuries to the eye as a result of laser exposure. Braynova et al. [3] also reported cases of severe burns as a result of laser hair removal that needed quick medical attention to prevent further adverse physical and psychological effects. In our case, the technician was treated with intravitreal anti-VEGF after the development of subfoveal CNV. This treatment was mandatory for the control of neovascularization and limitation of visual loss.

Jbara et al. [4] reported a case of accidental macula damage caused by a cosmetic laser during alignment. Even in such short contact time, severe visual damage can be accrued. Our case also illustrates the very rapid evolution from the insult to significant visual decline, requiring prompt medical intervention.

Diafas et al. [5] described CNV following an accidental alexandrite laser injury in which urgent intravitreal anti-VEGF treatment was given to prevent vision loss. Such a case, as well as ours, reiterates the message that early diagnosis and prompt treatment of CNV will ensure better visual outcomes.

Conclusively, ocular injuries from laser hair depilation are significant and can be prevented with safety measures and adequate training. In this case, the fact that a technician so young experienced tremendous damage to the retina leading to CNV further emphasizes the importance of maintenance of safety measures and early intervention. By describing this unique case, we hope to add to the information available in the management of such injuries and re-emphasize the importance of preventive measures to protect ocular health during cosmetic laser procedures.

## Conclusions

Ocular injuries that are caused by laser hair removal can result in severe, and at times irreversible, vision loss. The use of protective eyewear and adherence to safety guidelines is utmost in the prevention of these types of injuries. Immediate and proper medical intervention becomes important for reducing the damage and saving vision. Our case brings in the need for stringent safety measures with proper training being imparted to the personnel handling laser equipment on a regular basis. This is indeed an important case to contribute toward knowledge in the management and timely treatment of laser-induced ocular injuries for their prevention.
